# The hidden toll of colleague absenteeism: exploring its impact on emotional strain and burnout among frontline health workers in Nigeria

**DOI:** 10.3389/fpsyg.2026.1768286

**Published:** 2026-04-17

**Authors:** Onochie C. Eze, Iheomimichineke Ojiakor, Aloysius Odii, Pamela Adaobi Ogbozor, Izuchukwu Ndukaihe, Martin McKee, Dina Balabanova, Obinna Onwujekwe

**Affiliations:** 1 Health Policy Research Group, University of Nigeria Enugu Campus, Enugu, Nigeria; 2 Department of Sociology and Anthropology, University of Nigeria, Nsukka, Nigeria; 3 Department of Psychology, Enugu State University of Science and Technology, Enugu, Nigeria; 4 Department of Psychology, Alex Ekwueme Federal University, Ndufu-Alike, Abakaliki, Nigeria; 5 London School of Hygiene & Tropical Medicine, London, United Kingdom; 6 Department of Health Administration and Management, University of Nigeria Enugu Campus, Enugu, Nigeria

**Keywords:** burnout, emotional wellbeing, health worker absenteeism, primary healthcare centers, psychological strain

## Abstract

**Background:**

Absenteeism among health workers is a persistent challenge in resource-constrained health systems, increasing workloads for those who remain present and undermining service quality. While its operational consequences are well documented, the emotional and psychological impact on frontline workers remains underexplored. This study examines how unscheduled colleague absenteeism contributes to emotional strain and burnout among primary healthcare (PHC) workers in Nigeria.

**Methods:**

The study was conducted in Enugu and Kano States, using 24 in-depth interviews with PHC workers purposively selected from urban, semi-urban, and rural facilities, based on their direct experience with colleague absenteeism. Participants included both women and men, reflecting variation in workforce composition across settings. A multi-stakeholder co-creation workshop provided contextual and institutional perspectives. Data were analyzed thematically using the job demands-resources (JDR) framework, with interview data as the primary basis for analysis.

**Findings:**

Colleague absenteeism substantially increased job demands, with participants describing emotional exhaustion from repeatedly covering multiple clinical and non-clinical roles or managing facilities alone. These experiences generated significant emotional strain, manifesting as frustration, isolation, sadness, and moral distress. Effects were shaped by organizational conditions, including weak supervision, inconsistent shift management, and perceived unfairness linked to favoritism and limited accountability. Informal power structures, such as godfatherism, allowed some staff to be absent without consequence, while others assumed additional responsibilities, thereby intensifying perceptions of inequity. Gendered expectations further amplified emotional burden in settings where the PHC workforce was predominantly female.

**Conclusion:**

Unscheduled colleague absenteeism has profound emotional and psychological consequences for frontline PHC workers, contributing to burnout and diminished morale. Addressing these impacts requires workforce strategies that extend beyond staffing numbers to include transparent scheduling, supportive supervision, recognition of emotional labor, equitable accountability mechanisms, and gender-sensitive policies that prioritize the well-being of those who consistently remain present.

## Introduction

Health worker absenteeism remains a persistent challenge in many health systems, particularly in low- and middle-income countries (LMICs), where limited resources and fragile workforce structures already constrain service delivery. Across such settings, the absence of even a single health worker can disrupt workflow, increase patient waiting times, and compromise the quality and continuity of care (Onwujekwe et al., 2019; Agwu et al., 2020). Although absenteeism has long been recognized as a governance and accountability concern, growing pressures on frontline health systems have heightened its contemporary relevance. Chronic workforce shortages, high patient volumes, and weak supervision structures intensify the strain on health workers who remain present when colleagues are absent (Kwon, 2020; Odii et al., 2022; Ogbozor et al., 2022). At the same time, global attention to the mental health and wellbeing of health workers has increased, particularly following recent health system shocks and the growing recognition that workforce wellbeing is central to health system resilience and sustainability (World Health Organization, 2024).

While existing research has documented the prevalence, drivers, and governance implications of health worker absenteeism, far less attention has been paid to how the absence of colleagues affects the emotional and psychological wellbeing of those who remain on duty. Much of the literature focuses on operational consequences, such as reduced productivity, disrupted service delivery, and weak accountability structures (Onwujekwe et al., 2019; Obodoechi et al., 2021; Odii et al., 2022). Yet in facilities where staffing levels are already fragile, the absence of a colleague often requires remaining workers to absorb additional clinical and administrative responsibilities, potentially intensifying workload, emotional labor, and role conflict. Evidence from burnout research suggests that sustained exposure to such conditions is strongly associated with emotional exhaustion, stress, and reduced professional wellbeing among health workers (Khammissa et al., 2022; Ndulue et al., 2024). Despite these insights, the specific emotional consequences of colleague absenteeism remain insufficiently examined, particularly in primary healthcare (PHC) settings in LMICs.

To better understand these dynamics, this study draws on the job demands-resources (JDR) framework (Demerouti et al., 2001). As detailed in the theoretical framework below, this model examines how excessive job demands and eroded resources drive emotional exhaustion and burnout (Bakker and Demerouti, 2007).

Nigeria's primary healthcare (PHC) system serves as the empirical setting for this study. As detailed in the literature review, its structural characteristics, including chronic workforce shortages, weak supervision, informal power structures, and a predominantly female frontline workforce, make it a compelling context for examining how colleague absenteeism generates emotional strain. In summary (World Health Organization, 2024; Onwujekwe et al., 2019; Odii et al., 2022; Ogbozor et al., 2022; Nilsen et al., 2017; Morgan et al., 2022).

Against this background, this study examines how unscheduled colleague absenteeism contributes to emotional strain and burnout among primary healthcare workers in Nigeria. Drawing on qualitative evidence from frontline workers in Enugu and Kano States, the study explores how absenteeism reshapes daily work experiences, redistributes responsibilities, and generates psychological stress for those who remain present. By applying the job demands-resources framework to analyze these experiences, the study contributes to existing scholarship in three ways. First, it reframes absenteeism not only as a governance or managerial issue but also as a significant source of emotional stress embedded in everyday clinical practice. Second, it provides empirical insights into how organizational conditions, informal accountability structures, and gendered labor expectations shape the emotional consequences of absenteeism in resource-constrained health systems. Third, the study highlights the importance of recognizing and addressing the psychological wellbeing of frontline health workers as part of broader efforts to strengthen workforce resilience and sustain primary healthcare delivery.

## Theoretical framework

The job demands-resources (JDR) model provides the theoretical framework for this study. It posits that employee wellbeing is shaped by the interaction between job demands and job resources, operating through two processes: a health impairment pathway in which excessive demands lead to exhaustion, and a motivational pathway in which adequate resources foster engagement and resilience (Demerouti et al., 2001). This dual-process structure makes the JDR framework well-suited to healthcare settings, where emotional and workload pressures coexist with varying levels of institutional support.

Colleague absenteeism can function as a demand-amplifying mechanism within this framework. When a staff member is absent, remaining workers absorb additional clinical and administrative responsibilities, often without formal redistribution, increasing workload intensity, role ambiguity, time pressure, and emotional labor. Sustained exposure to such demands in emotionally intensive occupations has been consistently linked to exhaustion and burnout (Khammissa et al., 2022; Ndulue et al., 2024). Absenteeism may simultaneously erode job resources: when it disrupts team cohesion or is tolerated through informal power structures, perceptions of fairness and organizational support diminish, and inconsistent accountability further constrains workers' capacity to cope. Within the JDR model, this simultaneous increase in demands and reduction in resources accelerates the health impairment process.

The framework has been widely applied to burnout research across nursing, community health, and hospital settings (Khammissa et al., 2022; Saei et al., 2024), and its flexibility allows for context-specific identification of demands and resources while maintaining theoretical coherence. In fragile health systems where governance conditions shape daily practice, it offers a structured lens for linking organizational dynamics to individual psychological outcomes. This study applies the JDR model to position absenteeism not merely as a governance failure but as a structural condition that reshapes the demands–resources balance in everyday clinical work, with consequences for emotional strain and burnout among frontline PHC workers in Nigeria.

Job demands are the physical, emotional, or psychological aspects of a job that require sustained effort and are associated with certain costs, such as fatigue, stress, or burnout. In the context of healthcare, for example, job demands may arise from increased patient loads, long working hours, or the emotional toll of caregiving. The JDR model posits that these demands are primarily linked to the exhaustion component of burnout, which is characterized by feelings of being overextended and emotionally drained.

On the other hand, job resources are the physical, psychological, social, or organizational aspects of a job that help employees achieve work goals, reduce job demands, and foster personal growth. In healthcare settings, job resources involve supportive management, flexible scheduling, or community involvement. The model emphasizes that job resources are primarily associated with engagement and motivation and serve as a buffer against the negative effects of job demands. When resources are sufficient, they can mitigate the risk of burnout and enhance employee wellbeing.

The JDR model operates through two interconnected processes. The first is the health impairment process, where excessive job demands lead to physical and emotional exhaustion, ultimately resulting in burnout. The second is the motivational process, where the availability of job resources enhances engagement and resilience, enabling employees to cope with demands and maintain high performance (Demerouti et al., 2001). These processes highlight the dual pathways through which workplace conditions influence employee wellbeing and morale.

## Health system and PHC workforce context

Nigeria's primary healthcare (PHC) workforce comprises nurses, community health extension workers (CHEWs), community health officers (CHOs), and other facility-based staff who operate side by side within the same facilities. Although these cadres differ substantially in their formal training, regulatory oversight, and scope of clinical authority, the day-to-day realities of service delivery often lead to role overlap in practice. Nurses and CHOs are trained to undertake a wider range of clinical tasks, including maternal and newborn care, minor treatments, and supervisory responsibilities. CHEWs, by contrast, receive shorter, community-oriented training focused on health promotion, basic diagnostics, and routine outpatient services. In practice, informal task shifting often occurs without supportive supervision or oversight to cope with staff shortages and absenteeism.

Despite these differences, chronic understaffing and high patient demand have led to informal task shifting, with CHEWs frequently performing duties beyond their formal scope, including conducting deliveries, administering certain medications, and independently managing a facility when more clinically trained staff are absent. Conversely, nurses and CHOs also take on tasks considered foundational to community-based care, including outreach, documentation, and facility maintenance. Thus, while cadres are not formally interchangeable, the functional boundaries between them are routinely blurred in everyday practice, creating a shared pool of responsibilities that must be redistributed when a team member is absent.

## Literature review

### Health worker absenteeism in health systems

Health worker absenteeism has been widely documented as a persistent challenge in health systems, particularly in low- and middle-income countries (LMICs). Studies across Africa and other resource-constrained settings show that absenteeism undermines service delivery, increases waiting times, and reduces the quality and continuity of care (Onwujekwe et al., 2019; Agwu et al., 2020). In many contexts, absenteeism reflects deeper institutional weaknesses, including poor remuneration, limited career progression, inadequate supervision, and weak accountability mechanisms (Kwon, 2020; Odii et al., 2022; Ogbozor et al., 2022). Where governance structures are fragile, informal practices such as dual practice, political patronage, and kinship networks may further enable staff absence without sanction (Onwujekwe et al., 2020; Odii et al., 2022).

Research in Nigeria has similarly documented high levels of absenteeism in primary healthcare facilities and identified a range of structural and organizational drivers. These include low salaries, delayed payments, weak supervision, and limited enforcement of attendance rules (Agwu et al., 2020; Obodoechi et al., 2021). In such environments, absenteeism becomes normalized as part of everyday organizational practice, reflecting broader governance challenges within the health system (Onwujekwe et al., 2019). Although this body of literature has generated important insights into the causes and institutional dynamics of absenteeism, its primary focus has been on operational consequences, such as productivity losses, service disruptions, and governance failures. Much less attention has been paid to how absenteeism affects the emotional experiences of those health workers who remain present.

### Burnout and emotional labor in healthcare work

Burnout has been widely recognized as a critical challenge affecting health workers globally. Maslach and Jackson's seminal work conceptualizes burnout as a psychological syndrome characterized by emotional exhaustion, depersonalization, and reduced personal accomplishment arising from chronic workplace stress (Maslach and Jackson, 1981). Subsequent research has demonstrated that burnout is particularly prevalent among healthcare workers due to the emotionally demanding nature of patient care, heavy workloads, and exposure to human suffering (Schaufeli and Taris, 2014; Khammissa et al., 2022). High levels of burnout among health workers have been linked to decreased job satisfaction, reduced quality of care, increased medical errors, and higher turnover intentions (West et al., 2018).

Closely related to burnout is the concept of emotional labor, which refers to the requirement that workers manage and regulate their emotions as part of their professional roles (Hochschild, 2012). In healthcare settings, emotional labor is central to caregiving, as health workers must continually display empathy, compassion, and emotional stability while dealing with stressful or emotionally demanding situations. A growing body of research demonstrates that sustained emotional labor can contribute significantly to emotional exhaustion and burnout, particularly when organizational support is limited (Jeung et al., 2018; Saei et al., 2024). In resource-constrained health systems, where workloads are high and support structures are weak, these emotional demands may be even more pronounced.

### Gendered dimensions of health workforce stress

Gender dynamics play an important role in shaping experiences of stress and burnout within the health workforce. Globally, women constitute a large proportion of frontline healthcare workers, particularly in nursing and community health roles (Boniol et al., 2019). In many contexts, women health workers face a “double burden” of professional responsibilities and unpaid domestic care work, which can intensify work–life conflict and psychological strain (Nilsen et al., 2017; Nyberg et al., 2021). Studies have shown that women health workers often experience higher levels of emotional exhaustion and psychological distress than their male counterparts, partly due to these overlapping responsibilities and gendered expectations of caregiving (Morgan et al., 2022).

Evidence from recent health crises has further highlighted these gendered disparities. For example, research during the COVID-19 pandemic found that female healthcare workers reported significantly higher levels of anxiety, depression, and insomnia compared to male colleagues (Liu et al., 2021; Hennein et al., 2023). These findings suggest that workplace stressors may interact with broader social and gender norms to shape health workers' emotional experiences. In health systems where frontline cadres are predominantly female, such dynamics may amplify the psychological consequences of organizational challenges, including staffing shortages and increased workload.

### Absenteeism as a potential emotional stressor

Although burnout and emotional labor have been extensively studied in healthcare settings, the role of colleague absenteeism as a specific source of emotional strain has received relatively limited attention. When staffing levels are already low, the absence of a colleague may require remaining workers to take on additional responsibilities, extend working hours, or perform tasks beyond their formal roles. Such conditions can increase workload intensity, create role ambiguity, and disrupt teamwork, all of which are recognized predictors of burnout in healthcare environments (Schaufeli and Taris, 2014; Khammissa et al., 2022).

Organizational justice theory provides further insight into how absenteeism may generate emotional distress. When staff perceive that attendance rules are applied inconsistently or that some workers are protected from sanctions through informal power relations, perceptions of fairness and organizational trust may deteriorate (Colquitt, 2005). Similarly, psychological contract theory suggests that when expectations of shared responsibility and reciprocity within the workplace are violated, workers may experience frustration, disengagement, and reduced organizational commitment (Rousseau, 2004; Cassar and Buttigieg, 2015). In this way, absenteeism redistributes not only tasks but also emotional labor among remaining workers.

Despite these theoretical insights, empirical research explicitly linking colleague absenteeism to emotional strain and burnout among frontline health workers remains limited, particularly in LMIC primary healthcare settings. This represents an important gap in the literature. By examining how unscheduled colleague absenteeism shapes the emotional experiences of primary healthcare workers in Nigeria and analyzing these experiences through the job demands-resources framework, this study contributes new evidence on the hidden psychological consequences of absenteeism within fragile health systems.

## Methods

### Study design and setting

This study employed a qualitative research design using in-depth interviews to explore the emotional and psychological effects of colleague absenteeism among primary healthcare (PHC) workers in Nigeria. The study formed part of the anti-corruption evidence (ACE) research program, which examines governance challenges, accountability dynamics, and informal practices within Nigeria's health system. The research was conducted in Enugu State in southeastern Nigeria and Kano State in northwestern Nigeria. These states were purposively selected to capture variation across geopolitical zones and socio-cultural contexts within the Nigerian health system. Within each state, local government areas (LGAs) were selected based on patterns of absenteeism identified during earlier phases of the ACE project and to ensure representation of urban, semi-urban, and rural PHC settings.

In Enugu State, five LGAs (Enugu North, Enugu South, Enugu East, Nsukka, and Igbo-Etiti) were selected, and 12 PHC facilities were included to capture variation across urban, semi-urban, and rural contexts. In Kano State, nine LGAs (Kura, Kumbotso, Nasarawa, Gwale, Tarauni, Fagge, Dala, Ungogo, and Kano Municipal) were selected, with six PHC facilities included in the study. Facilities were purposively selected to capture variation in staffing patterns, supervisory practices, and organizational environments that may shape experiences of absenteeism.

This purposive sampling approach aimed to capture diverse experiences of colleague absenteeism across different facility contexts and professional roles rather than to achieve statistical representation. The approach allowed in-depth exploration of the lived experiences of frontline health workers while enabling comparison across settings.

### Participants

Twenty-four health workers participated in in-depth interviews. In Enugu State, 12 female health workers were purposively selected from 12 PHCs across five LGAs, including nurses, CHEWs, and OICs, aged 31 to 53 years, with varying levels of education and experience. PHCs were located in urban (Ogui, Abakpa, Asata, Nsukka), semi-urban (Ngwo Hilltop, Emene, Ibagwa Nike, Amechi Awkunanaw), and rural (Agbani, Opi, Ogbede, Aku) settings.

In Kano State, 12 PHC workers were purposively selected from six PHCs, including nurses, CHOs, pharmacy technicians, and senior medical officers. All participants had direct experience with colleague absenteeism and were willing to discuss its effects.

### Analytical rationale for cross-cadre analysis

Although the cadres represented in this study differ in training and formal scope, they are analyzed collectively because chronic understaffing routinely blurs functional boundaries in everyday practice. Absenteeism therefore, does not merely remove a specific skill set; it redistributes overlapping duties among all remaining staff regardless of cadre. All frontline workers are drawn into the same cycles of covering for absent colleagues, absorbing additional tasks, and sustaining service delivery with limited support. The PHC system's heavy reliance on CHEWs, who constitute the numerical backbone of frontline services and frequently serve as the first point of patient contact, further reinforces this integrated analytic approach.

A separate co-creation workshop was held in Enugu (January 2025) as part of the ACE2 Health Systems Project. This brought together 28 stakeholders: local government health authorities, PHC management boards, Ward Development Committee members, informal health providers, OICs, and researchers. It was designed to complement the interview data by testing the clarity and coherence of emerging themes, exploring alternative interpretations, and refining the meanings that participants attributed to key concepts. While the workshop did not serve as a primary data source for the psychological outcomes analyzed in this study, it provided valuable contextual insights into organizational practices, supervisory dynamics, and community expectations that shaped absenteeism experiences within facilities. The workshop discussions, therefore, served as a mechanism for validating and deepening interpretations derived from the interviews, ensuring that thematic analysis reflected both individual accounts and the broader institutional environment in which they were situated.

Socio-demographic characteristics of each group, interview participants and co-creation workshop participants, including gender, cadre, age range, years of experience, and facility location, are summarized in Supplementary Appendix 1 (Supplementary Tables 1, 2) to provide transparency of the sampling strategy.

### Data collection

Data were collected between June 2024 and January 2025 through in-depth interviews and a co-creation workshop. A semi-structured interview guide was developed iteratively, drawing on existing literature, prior phases of the ACE project, and the research team's expertise in absenteeism research. It explored participants' experiences of colleague absenteeism, emotional responses, coping strategies, and perceptions of supervision relating to routine experiences rather than time-specific events. The interview guide is included in the Supplementary Appendix 2 to enhance transparency and support reproducibility of the qualitative methods, consistent with good practice in reporting research findings. Topics included perceptions of absenteeism, emotional impacts, workload management, coping mechanisms, and effects of supervision and community involvement.

Interviews were conducted in English, Igbo, or Hausa and lasted 40 to 60 min. Sessions were audio-recorded with informed consent and transcribed verbatim. Field notes captured contextual details and non-verbal cues. Data collection continued until thematic saturation, with no new themes emerging by the 24th interview. The sample size captured variation across study sites and professional roles while allowing in-depth analysis.

Workshop data were collected through field notes and audio recordings of group discussions on causes, coping strategies, and interventions to reduce absenteeism. Workshop insights supported the completeness of identified themes.

No major policy changes, strikes, or health system shocks occurred during data collection that would alter absenteeism patterns or emotional responses, thereby allowing consistent capture of routine service-delivery experiences without temporal bias.

### Reflexivity

The research team had prior experience studying health systems and absenteeism in Nigeria and acknowledged that this could influence interpretation. To minimize bias, interviews were conducted by researchers not involved in facility management, multiple team members independently coded transcripts, and disagreements were resolved through consensus. Ongoing reflexive discussions were used to ensure analyzes remained grounded in participants' accounts. This methodological rigor, triangulation across multiple data sources, and theoretical integration provided a comprehensive understanding of how absenteeism disrupts service delivery, increases emotional strain, and contributes to burnout among frontline health workers in Nigeria.

### Data analysis

Data were analyzed thematically using NVivo 12 qualitative data analysis software (QSR International Pty Ltd., Burlington, MA, USA). Three researchers developed and refined a codebook through consensus to ensure validity and reliability. Each researcher independently coded transcripts, followed by consensus meetings to refine and merge overlapping codes. Constant comparative methods were used to identify patterns and variations across participants, locations, and cadres, thereby strengthening the coherence and depth of themes within the thematic analysis. Member checking with a subset of participants validated the emergent themes. An overview of analytic themes, sub-themes, and illustrative quotes derived from the interview data is presented in Supplementary Appendix 1 (Supplementary Table 3) to enhance transparency of theme development.

**Figure 1:**
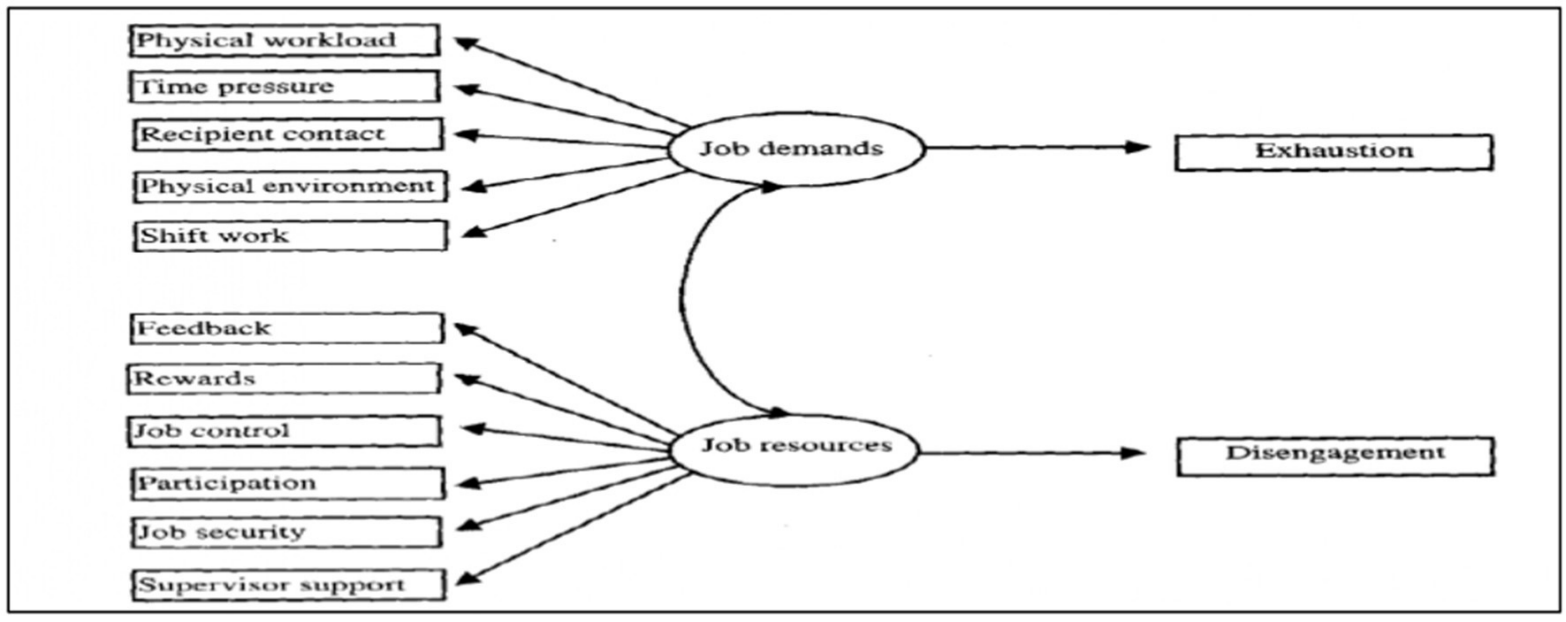
The job-demand resources (JDR) model (Demerouti et al., 2001).

Using the JDR model (Figure 1), the analysis examined how colleague absenteeism heightened job demands (increased workload, informal task shifting, role overload) and how job resources (supervisory support, teamwork, community engagement) shaped workers' emotional experiences and vulnerability to burnout. Workshop insights were incorporated solely to contextualize interview findings within broader organizational and institutional dynamics and to inform the identification of feasible, practice-relevant strategies, not to drive theme generation.

**Figure 2:**
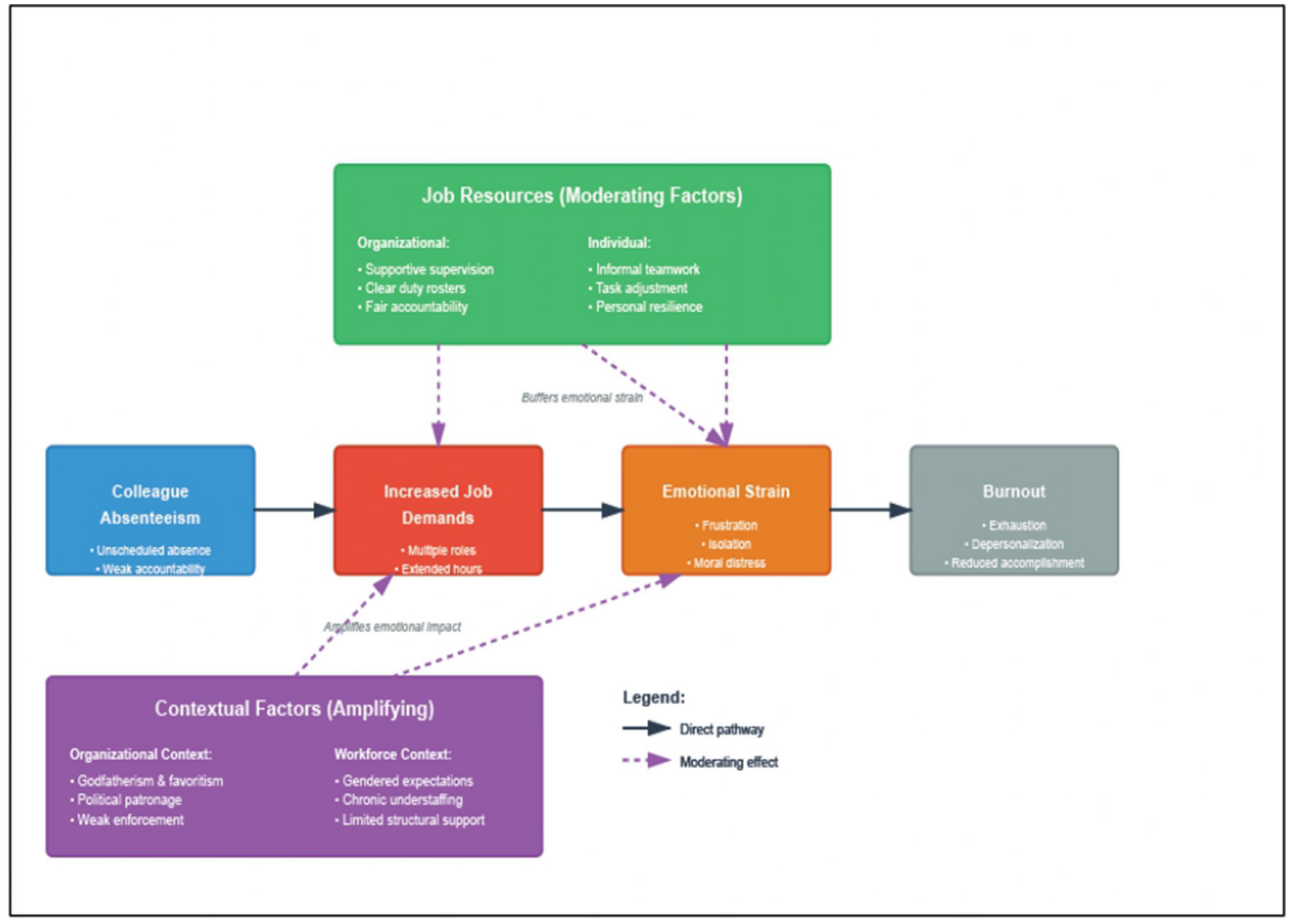
Conceptual model linking colleague absenteeism to burnout in primary healthcare workers (based on the JDR framework).

Guided by the JDR framework, Figure 2 (Supplementary Appendix 1) illustrates how colleague absenteeism increases job demands, generates emotional strain, and leads to burnout, with job resources moderating these effects.

## Findings

The findings are organized around three connected areas that show how colleague absenteeism creates emotional distress: (1) what triggers emotional responses, (2) the emotional outcomes experienced, and (3) the resources and coping strategies employed by health workers. This structure highlights the psychological impact of absenteeism while staying consistent with the job demands-resources framework. Insights from the co-creation workshop help explain organizational dynamics but are not used as primary data.

### Emotional triggers: conditions that generated emotional distress

Colleague absenteeism triggered emotional distress by increasing job demands and disrupting teamwork. Participants described covering multiple clinical and non-clinical roles, extending working hours, and sometimes managing facilities alone. These conditions were experienced as persistent stressors rather than temporary inconveniences.

“You don't expect midwives who stayed the whole day and helped in delivering at least five babies at night to continue working the next morning.” (CHEW, Enugu East)

Absenteeism also triggered distress when colleagues failed to provide explanations for their absence, creating uncertainty and resentment.

“At times, maybe when the person didn't explain the reason for being absent, it makes me feel bad.” (CHEW, Enugu East)

Informal task shifting and unclear shift arrangements further intensified distress by creating role ambiguity and anxiety.

“We are supposed to have division of labor, but I am taking care of all of them.” (CHEW, Enugu East)

In some facilities, absenteeism led workers to provide services alone for extended periods, resulting in feelings of isolation.

“Sometimes you are alone in the facility the whole day.” (CHEW, Enugu)

### Emotional outcomes: psychological consequences of absenteeism

These conditions produced a range of emotional and psychological outcomes. Emotional exhaustion emerged as the most pervasive, with participants describing feeling drained, overwhelmed, and unable to recover between shifts.

“When the staff are around at the right time, the work goes smoothly, but if they are absent, the work becomes too much to handle.” (Male OIC, Kano)

Participants also reported frustration and sadness, particularly when absenteeism was perceived as unexplained, unfair, or tolerated without consequences.

“I am usually sad by the absence of a colleague, especially when I don't know their reason.” (Male Pharmacy Technician, Kano)

When absenteeism was protected by favoritism or godfatherism, participants experienced moral distress from being compelled to compensate for colleagues' absences without the authority to challenge these practices.

“It is really painful if a colleague refuses to come to work as you should do his work and one will stress himself.” (Male CHO, Kano)

Some participants downplayed their emotional reactions:

“I don't feel anything by the presence or absence of my co-worker… even though it may affect the services I offer.” (Female Nurse, Kano)

Rather than indicating emotional indifference, such responses reflected coping strategies shaped by professional norms and power asymmetries that discouraged overt expression of distress.

### Supportive resources and strategies to manage emotional distress

Participants described several resources and strategies that helped them cope with emotional strain, though these were often limited or inconsistently applied. Informal teamwork and peer support were frequently relied upon to manage increased demands.

“I feel happy whenever my colleagues are present because working as a team makes the work easier.” (Female CHEW, Kano)

Supervisory oversight and clear duty rosters were described as important organizational resources that promoted accountability and reduced emotional strain.

“Knowing that there will be supervision makes workers always be there.” (CHEW, Enugu East)

Community support, particularly through Ward Development Committees, also helped mitigate emotional distress by fostering shared responsibility and recognition.

“The community members have a committee, so they contribute from time to time.” (CHEW, Enugu East)

However, many coping strategies, including emotional self-regulation, extended shifts, and endurance, were described as unsustainable, masking deeper systemic problems and contributing to longer-term burnout.

Policy-relevant implications arising from the findings are synthesized in Supplementary Appendix 1 (Supplementary Table 4), which presents tiered recommendations at the facility, state/LGA, and national levels.

## Discussion

This study examined the emotional and psychological consequences of colleague absenteeism among frontline primary healthcare (PHC) workers in Nigeria. Drawing on qualitative accounts from health workers in Enugu and Kano States, the findings show that absenteeism is experienced not merely as an operational disruption but as a significant emotional burden for those who remain present. Participants repeatedly absorbed additional responsibilities, managed facilities alone, and navigated uncertainty around colleagues' absence, conditions that generated emotional exhaustion, frustration, sadness, and moral distress.

These findings extend prior studies (Onwujekwe et al., 2019; Odii et al., 2022; Obodoechi et al., 2021) beyond their predominant focus on the operational and governance consequences of absenteeism. Where earlier studies concentrated on service delivery disruptions and institutional accountability failures (Agwu et al., 2020; Onwujekwe et al., 2020), this study reveals the psychological costs borne by workers who remain present. The study's specific contributions to knowledge are detailed in the dedicated section below.

The findings illustrate how colleague absenteeism intensified job demands within already resource-constrained facilities. Participants described covering additional clinical and administrative tasks, extending working hours, and managing patient loads without adequate support. These experiences align with the job demands-resources model, which suggests that excessive job demands combined with limited organizational resources increase vulnerability to emotional exhaustion and burnout (Bakker and Demerouti, 2007). In the present study, absenteeism served as a demand-amplifying condition that simultaneously eroded key resources, including teamwork, predictable scheduling, and supervisory support. When these resources were weak or inconsistently applied, workers experienced heightened stress and diminished emotional resilience.

These findings also resonate with prior studies on burnout in healthcare settings (Khammissa et al., 2022; Schaufeli and Taris, 2014; West et al., 2018). Previous studies have consistently linked heavy workloads, role conflict, and insufficient organizational support to emotional exhaustion among health workers (Khammissa et al., 2022). However, the present study highlights how absenteeism can act as a specific trigger for these conditions in primary healthcare facilities where staffing levels are already fragile. Rather than representing isolated incidents, participants described absenteeism as a recurring feature of their working environment, creating persistent uncertainty and redistributing responsibilities among remaining staff. Such conditions can gradually erode morale and contribute to cumulative psychological strain.

Beyond workload pressures, the findings also highlight the importance of organizational justice in shaping emotional responses to absenteeism. Participants frequently expressed frustration when colleagues' absences went unexplained or when attendance norms were applied unevenly. These perceptions align with organizational justice theory, which emphasizes the role of fairness and transparency in maintaining trust and commitment within organizations (Colquitt, 2005). Where some workers were perceived to be protected by informal power structures such as patronage or favoritism, remaining staff experienced moral distress from being required to compensate for absences without the authority to challenge these practices. In such contexts, absenteeism redistributes not only tasks but also emotional labor, potentially weakening team cohesion and workplace trust.

Gender dynamics further shaped these experiences. Women constituted the majority of participants and described navigating overlapping professional and domestic responsibilities while coping with increased workload pressures. This finding reflects broader evidence that women health workers often experience compounded stress due to the intersection of paid employment and unpaid caregiving roles (Morgan et al., 2022). In contexts where the frontline workforce is predominantly female, expectations that women will absorb additional responsibilities without complaint may intensify emotional strain. The accounts in this study suggest that absenteeism-related burdens may interact with gender norms to amplify the risk of burnout among women health workers.

Participants also identified resources that helped mitigate emotional strain, although these were often limited or inconsistently applied. Informal teamwork, peer support, and clear supervisory oversight were described as important mechanisms for managing workload pressures and maintaining morale. These findings reinforce evidence that supportive organizational environments can buffer the negative effects of job demands on worker wellbeing (Bakker and Demerouti, 2007). However, many coping strategies described by participants, including emotional self-regulation, extended working hours, and silent endurance, were ultimately unsustainable and masked deeper systemic problems related to staffing shortages and weak accountability mechanisms.

Taken together, these findings suggest that absenteeism operates as a systemic stressor within fragile health systems. When attendance norms are inconsistently enforced and institutional support is weak, the emotional burden of absenteeism is redistributed downward to frontline workers who remain present. Over time, such dynamics may contribute to burnout, declining morale, and workforce attrition, challenges that are already widely recognized in LMIC health systems (World Health Organization, 2024).

Ultimately, this study centers on the experiences of health workers who continue to show up and sustain service delivery despite persistent institutional challenges. Recognizing and addressing the emotional consequences of absenteeism is therefore essential not only for improving workforce wellbeing but also for strengthening the resilience and sustainability of primary healthcare systems in resource-constrained settings.

### Contribution to knowledge and implications for practice

This study advances existing knowledge on health worker absenteeism in three interconnected ways. First, it reframes absenteeism as a psychological stressor for present workers, not merely an operational or governance problem, showing empirically that it generates emotional exhaustion, frustration, sadness, isolation, and moral distress among those who remain on duty. Second, applying the job demands-resources framework reveals the mechanism: absenteeism simultaneously intensifies job demands and erodes key resources such as teamwork, predictable scheduling, and supervisory oversight, thereby accelerating the health impairment pathway to burnout. Third, the study deepens understanding of how power dynamics and organizational justice shape the emotional intensity of these experiences: informal practices such as godfatherism shield some staff from accountability while redistributing not only tasks but also emotional labor onto those who remain, a dynamic further amplified by gendered expectations in predominantly female workforces.

These findings have practical implications that move beyond traditional monitoring or disciplinary approaches. Strengthening clarity and fairness in scheduling, ensuring consistent supervisory support, and fostering a culture that recognizes emotional labor are all essential for reducing strain on present workers. Improving accountability for attendance can help rebuild organizational trust, while attention to gendered pressures and the psychological well-being of frontline staff is crucial for sustaining motivation and preventing burnout. By showing how absenteeism is experienced both emotionally and operationally, the study underscores the need for workforce strategies that value presence, ensure fairness, and support the psychological health of those who keep primary healthcare systems functioning.

### Study limitations

This study has several limitations that should be considered when interpreting the findings. First, the study employed purposive sampling of frontline health workers with direct experience of colleague absenteeism. While this approach enabled in-depth exploration of participants' lived experiences, it may limit the transferability of the findings to other health system contexts beyond similar primary healthcare settings in Nigeria.

Second, the data relied on participants' retrospective accounts of workplace experiences, which may be influenced by recall bias, particularly when describing emotionally salient events. However, the use of in-depth interviews and triangulation with workshop discussions provided richer contextual understanding and strengthened the credibility of the findings.

Third, the study used a cross-sectional qualitative design, capturing participants' experiences at a single point in time. As a result, the study cannot examine how emotional strain associated with absenteeism evolves or how workers' coping strategies may change in response to shifting organizational conditions. Longitudinal research could provide deeper insight into how sustained exposure to absenteeism influences burnout trajectories among frontline health workers.

Finally, the predominance of female participants reflects the gendered composition of the primary healthcare workforce in the study settings, particularly in Enugu State. While this provided valuable insight into gendered dimensions of emotional labor, future studies may benefit from exploring how experiences of absenteeism differ across gender, cadre, and career stage in a wider range of health system contexts.

Despite these limitations, the study provides important insights into an often overlooked dimension of health workforce challenges. By highlighting the emotional and psychological consequences of colleague absenteeism for frontline health workers, the findings contribute to a more comprehensive understanding of workforce wellbeing in resource-constrained health systems and underscore the need for organizational strategies that address both operational and psychological aspects of absenteeism.

## Conclusion

The accounts shared by health workers in this study illuminate what it means to remain present in Nigeria's primary healthcare facilities consistently. Beyond their formal responsibilities, these workers carry a significant emotional burden shaped by unscheduled colleagues' absences and the expectation that they will quietly absorb their consequences. Their narratives reveal how absenteeism is experienced not simply as a logistical disruption, but as a deeply personal strain that affects motivation, wellbeing, and professional commitment.

Participants described how colleague absenteeism translates into emotional exhaustion from repeated overextension, frustration and sadness when absences go unexplained, and moral distress when accountability is uneven or absent. These are not abstract or episodic challenges but cumulative experiences that erode morale, undermine psychological resilience, and threaten the sustainability of frontline care. Yet the emotional consequences of absenteeism remain insufficiently recognized within workforce-strengthening strategies, which often prioritize staffing numbers and service outputs over the wellbeing of those who remain on duty.

By centring the emotional experiences of health workers who continue to show up, this study underscores the need for policies and management approaches that go beyond disciplinary or staffing solutions. Addressing absenteeism effectively requires recognizing and supporting frontline workers' psychological health, ensuring fair and transparent accountability, and strengthening organizational environments in which supportive supervision, shared responsibility, and emotional wellbeing are integral to primary healthcare delivery.

## Data Availability

The raw data supporting the conclusions of this article will be made available by the authors, without undue reservation.
