# Supplementary material for: The hidden toll of colleague absenteeism: exploring its impact on emotional strain and burnout among frontline health workers in Nigeria
Source: Front Psychol. 2026 Apr 17;17:1768286. doi: 10.3389/fpsyg.2026.1768286 (PMC13132692; doi:10.3389/fpsyg.2026.1768286)
Supplement: Supplementary file 1 [file Table_1.docx]

# **Annex 1:** Tables

**Table 1.** Socio-demographic characteristics of interview participants (n = 24)

| **Characteristic** | **Category** | **n (%)** |
| --- | --- | --- |
| Gender | Female | 19 (79.2) |
|  | Male | 5 (20.8) |
| State | Enugu | 12 (50.0) |
|  | Kano | 12 (50.0) |
| Cadre | Community Health Extension Workers (CHEWs) | 14 (58.3) |
|  | Community Health Officers (CHOs) | 5 (20.8) |
|  | Nurses / Midwives | 4 (16.7) |
|  | Other PHC staff (e.g., pharmacy technician) | 1 (4.2) |
| Age range (years) | 31–40 | 8 (33.3) |
|  | 41–50 | 10 (41.7) |
|  | 51–53 | 6 (25.0) |
| Years of experience | 5–10 years | 6 (25.0) |
|  | 11–20 years | 11 (45.8) |
|  | >20 years | 7 (29.2) |
| **Facility location** | Urban | 10 (41.7) |
|  | Semi-urban | 7 (29.2) |
|  | Rural | 7 (29.2) |

**Table 2**. Socio-demographic characteristics of co-creation workshop participants (n = 28)

| **Characteristic** | **Category** | **n (%)** |
| --- | --- | --- |
| Gender | Female | 16 (57.1) |
|  | Male | 12 (42.9) |
| Participant type | PHC frontline health workers | 11 (39.3) |
|  | Facility managers / Officers-in-Charge (OICs) | 6 (21.4) |
|  | Local government health officials | 5 (17.9) |
|  | Community representatives (WDC members) | 4 (14.3) |
|  | Development partners / CSOs | 2 (7.1) |
| Years of experience | <10 years | 7 (25.0) |
|  | 10–20 years | 12 (42.9) |
|  | >20 years | 9 (32.1) |
| Primary role in PHC system | Service delivery | 13 (46.4) |
|  | Supervision / management | 9 (32.1) |
|  | Community governance / engagement | 6 (21.5) |

**Table 3.** Summary of themes, sub-themes, and illustrative quotes on colleague absenteeism

| **Broad theme** | **Sub-themes** | **Illustrative quotes** |
| --- | --- | --- |
| Emotional triggers | Increased workload and role overload | “You don’t expect midwives who stayed the whole day and helped in delivering at least five babies at night to continue working the next morning.” (CHEW, Enugu East) |
|  | Unexplained absenteeism | “At times, maybe when the person didn’t explain the reason for being absent, it makes me feel bad.” (CHEW, Enugu East) |
|  | Informal task shifting and role ambiguity | “We are supposed to have division of labour, but I am taking care of all of them.” (CHEW, Enugu East) |
|  | Isolation during service delivery | “Sometimes you are alone in the facility the whole day.” (CHEW, Enugu) |
| Emotional outcomes | Emotional exhaustion | “When the staff are around at the right time, the work goes smoothly, but if they are absent, the work becomes too much to handle.” (Male OIC, Kano) |
|  | Frustration and sadness | “I am usually sad by the absence of a colleague, especially when I don’t know their reason.” (Male Pharmacy Technician, Kano) |
|  | Moral distress and perceived unfairness | “It is really painful if a colleague refuses to come to work as you should do his work.” (Male CHO, Kano) |
|  | Emotional suppression as coping | “I don’t feel anything by the presence or absence of my co-worker.” (Female Nurse, Kano) |
| Supportive resources and coping Strategies | Informal teamwork and peer support | “I feel happy whenever my colleagues are present because working as a team makes the work easier.” (Female CHEW, Kano) |
|  | Supervision and accountability | “Knowing that there will be supervision makes workers always be there.” (CHEW, Enugu East) |
|  | Community support mechanisms | “The community members have a committee, so they contribute from time to time.” (CHEW, Enugu East) |
|  | Endurance and emotional self-regulation | “We just try to cope, because there is no option.” (CHEW, Kano) |

**Table 4:** Tiered policy recommendations to address health worker absenteeism and emotional Strain

| **Level** | **Key issues** | **Priority policy actions** |
| --- | --- | --- |
| Facility level (PHCs) | High workload, burnout, weak shift management | • Enforce transparent duty rosters  • Use supportive, non-punitive supervision  • Introduce peer support and routine debriefing  • Clarify task roles |
| State / LGA level | Understaffing, weak accountability, uneven leadership | • Recruit and equitably deploy staff  • Strengthen attendance monitoring  • Train supervisors in fair, supportive leadership • Activate Ward Development Committees |
| National level | Workforce shortages, gendered burden, limited well-being focus | • Integrate mental health into PHC workforce policy  • Issue national guidance on shift management and supervision  • Implement gender-sensitive measures  • Balance accountability with worker support |
