# Supplementary material for: The hidden toll of colleague absenteeism: exploring its impact on emotional strain and burnout among frontline health workers in Nigeria
Source: Front Psychol. 2026 Apr 17;17:1768286. doi: 10.3389/fpsyg.2026.1768286 (PMC13132692; doi:10.3389/fpsyg.2026.1768286)
Supplement: Supplementary file 2 [file Table_2.docx]

**Annex 2: Interview guide**

**Introduction**

This study seeks to find ways and means of increasing the absence of health workers and their productivity in primary health facilities in Enugu, Nigeria. Using a co-creation approach, the intervention will be designed to enhance the presence and commitment of health workers at the frontline.

**Demographic information of respondent:**

Designation (OIC or non-OIC):

Gender:

Age group: 1 = less than 30 years 2 = 30 to 50 years 3 = above 50 years [ ]

Highest level of education:

Length of service as a health worker (in years):

Location of the health facility (rural/semi-urban/urban):

**Interviews with not more than two present health workers which may include OIC (seek to record)**

1. What motivates you to come to work? (Check for reasons around duty, passion, managerial sanctions, living in facility, little stipends from service, revenue drive for facility, personal benefits etc.)
2. Would you have been at work today if you had the option of attending to other private businesses?
3. Are there some of your colleagues who are supposed to be at work today but are not in the health facility?
4. How does it make you feel that you are here, and they are not? (even if you find one person short, ask this question with that one missing person in mind)
5. Why do you think that your colleagues are absent from work today?

*(Check for transportation cost, negligence, lack of supervision, lack of commitment to attendance register, the use of volunteers as cover, private businesses, dual practices, lack of equipment, lack of living quarters, irregularity in employment due to politics and social connections, etc.)*

1. *Why do you think that your colleagues are sometimes absent from work?*
2. What strategies are in place to ensure staff report to work when scheduled?
   1. Among these strategies listed, which ones are effective and which are not?
      1. Please share an example of when the strategies were effective?
      2. What are the reasons why some of the strategies are not effective?
   2. What can be done so that your absent colleagues can be present?
3. What other benefits do health workers in your facility get apart from their salaries? (Probe: incentives, bonuses, non-financial incentives)
   1. What are the sources of the benefits?
   2. What are these benefits for?
4. Why do you think absenteeism (coming to work late, leaving before shift is over, leaving before the end of work and not coming to work for a whole day even when one is supposed to come) exists in Primary Health facilities?
5. What do you think are the best ways to curb absenteeism (coming to work late, leaving before shift is over, leaving before the end of work and not coming to work for a whole day even when one is supposed to come) in Primary Health facilities?
6. Why do you think these strategies for reducing absenteeism will work? (probe to see if the strategies given in no.10 have been tried out previously and why the respondent believes it did not work and how best they think it should be done this time for it to work).

Thank the respondent(s) for their time and cooperation.
